# Are You Approaching Me? Motor Execution Influences Perceived Action Orientation

**DOI:** 10.1371/journal.pone.0037514

**Published:** 2012-05-18

**Authors:** Valeria Manera, Andrea Cavallo, Claudia Chiavarino, Ben Schouten, Karl Verfaillie, Cristina Becchio

**Affiliations:** 1 Department of Psychology, Center for Cognitive Science, University of Turin, Turin, Italy; 2 Laboratory of Experimental Psychology, K.U. Leuven, Leuven, Belgium; Katholieke Universiteit Leuven, Belgium

## Abstract

Human observers are especially sensitive to the actions of conspecifics that match their own actions. This has been proposed to be critical for social interaction, providing the basis for empathy and joint action. However, the precise relation between observed and executed actions is still poorly understood. Do ongoing actions change the way observers perceive others' actions? To pursue this question, we exploited the bistability of depth-ambiguous point-light walkers, which can be perceived as facing towards the viewer or as facing away from the viewer. We demonstrate that point-light walkers are perceived more often as facing the viewer when the observer is walking on a treadmill compared to when the observer is performing an action that does not match the observed behavior (e.g., cycling). These findings suggest that motor processes influence the perceived orientation of observed actions: Acting observers tend to perceive similar actions by conspecifics as oriented towards themselves. We discuss these results in light of the possible mechanisms subtending action-induced modulation of perception.

## Introduction

Humans and non human primates are highly sensitive to where others are orienting their attention [1] and there is evidence that at both the behavioural and the neural level the processing of others' bodily movement is influenced by social attention cues such as gaze direction and head and body orientation [2]–[6]. For instance, response to actions of conspecifics in the superior temporal sulcus region has been shown to be stronger for actions oriented towards the observer (i.e., a walking person gazing at the observer) compared to actions oriented away (i.e., a walking person gazing away from the observer) [4]. A similar effect has also been reported for the activation of the neural network that is activated both during observation of an action and during execution of the same action, the so-called mirror neuron system [7], [8]. Using magnetoencephalography, it has been documented that the degree to which α-band oscillatory activity in the 7–12 Hz frequency range is attenuated during action observation – an index of mirror neuron activity – is dependent upon whether the agent is facing towards or away from the observer [9]. When the agent is facing the observer, the oscillations in the α-frequency range are relatively greater at parietal sensors contralateral, and relatively lower at parietal sensors ipsilateral, to the hand observed. When the actor is facing away there is no clear modulation in these parameter estimates. Similarly, a significant modulation in the β oscillation range (15–30 Hz) originating in the primary motor cortex is found when the agent is looking at and turned towards the observer, but not when agent is looking away [10].

These findings suggest that there is an important link between perceived body orientation and motor simulation processes. Considering that when someone has her/his back turned the social relevance of the action is much reduced compared with when she or he is facing the observer, it is perhaps not surprising that actions facing the viewer elicit a stronger motor resonance [9]. However, to our knowledge, no study so far has investigated the existence of an effect in the opposite direction, i.e., whether concurrent motor execution might influence the perceived orientation a given action. A direct link between perception and action, as implied by mirror mechanisms, predicts not only effects of perception on action but also effects of action on perception [11]. Just as the orientation of the perceived person influences motor activation in the observer, motor execution of the observer might thus be expected to influence the perceived orientation of the observed person.

**Figure 1:**
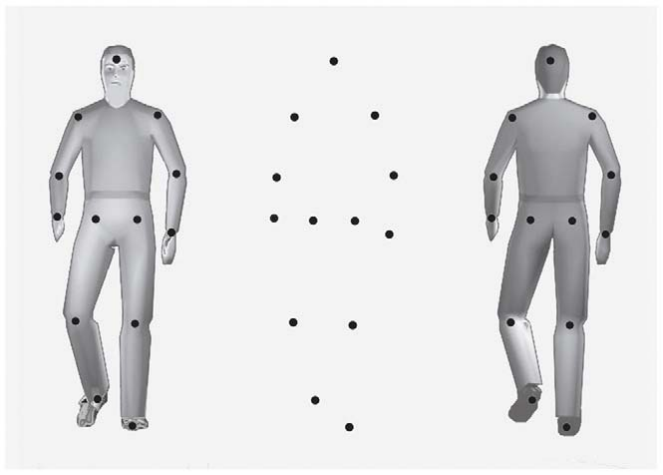
Illustration of a bistable point-light walker. Illustration of a single frame of the bistable point-light walker (without perspective cues), and the two veridical interpretations with the point-lights superimposed. Whilst both veridical interpretations are equally plausible, observers usually prefer the FV interpretation. Thus, the objectively bistable point-light walker does not correspond to the subjectively bistable one. To obtain *subjectively* bistable stimuli, we performed a preliminary adjustment task in which perspective cues carrying the information concerning the veridical orientation of the point-light figure were gradually manipulated. On each trial, participants were asked to indicate whether the visually presented stimulus was perceived as FV or FAV. Individual perspective levels were determined by fitting a cumulative Gaussian function to the proportion of FV responses in the different perspective conditions, and selecting the perspective manipulation corresponding to the 30%, 50%, and 70% FV thresholds.

Here we tested this hypothesis by using subjectively bistable point-light figures [12], [13]. Similar to the Schröder staircase and the Necker cube, bistable point-light displays are consistent with two in principle equally plausible 3D interpretations, which differ only with respect to the depth order of the body parts. For instance, a point-light walker in frontal view can be perceived as facing the viewer (FV) or facing away from the viewer (FAV; Fig. 1). We exploited this characteristic of point-light stimuli in order to investigate the interaction between motor simulation and perceived action orientation.

In Experiment 1 participants were asked to judge the in-depth orientation of bistable point-light walkers while walking themselves on a treadmill (walking condition) or remaining in front of the screen without moving (no-movement condition). We reasoned that if activation of the observer's motor system influences the perceived orientation of the observed action, then concurrent execution of the same action, i.e., walking, should increase the proportion of FV responses.

To rule out that the effect was due to general movement of the observer or movement direction rather than to motor simulation of the specific action of walking, we conducted two control experiments in which the participants' walking action was substituted by performing lateral steps (Experiment 2) and riding an exercise bike (Experiment 3). Performing lateral steps involves the same limbs, but does not match the point-light walking action. If perceived action orientation is specifically influenced by motor simulation, then no FV responses increase should be observed when performing lateral steps. Similarly, if matching between executed and observed action is critical for producing the effect, no increase in FV response should be observed for cycling, i.e., by an action implying the same movement direction as walking, but involving a different type of movement.

## Methods

### Experiment 1

#### Participants

Twenty-five undergraduate and graduate students from the University of Turin (11 male and 14 female, mean age = 23.4 years, age range 19–30) volunteered to take part in the experiment. All had normal or corrected-to-normal vision, had provided informed written consent, and were naïve with respect to the purpose of the study. The study (as well as Experiment 2 and 3) was approved by the Ethical Committee of the Faculty of Psychology of the University of Turin and was conducted in accordance with the ethical standards laid down in the 1964 Declaration of Helsinki.

#### Stimuli

Stimuli consisted of point-light figures with 13 markers indicating the head and the center of the major joints of a person (shoulders, elbows, wrists, hips, knees, and feet). All point-light figures were derived from the same 3D-coordinates of a human walker of which the translational component was removed [14]. As a result, the walker moved as if walking on a treadmill, in the center of the screen. The visual angle between the points attached to the head and the feet was about 5.7 deg. Each dot subtended about 0.14 deg. Each stimulus presentation lasted 3 s consisting of two walking cycles, each consisting of two steps. The starting posture in the walking cycle was randomized across trials. Stimuli were black against a grey background, and were presented using Presentation software (14.4 version, Neurobehavioral Systems) on a. 15.4-inch WXGA monitor (display resolution: 1280×800; refresh rate: 60 Hz).

The in-depth orientation of the point-light figures (FV *vs.* FAV) was manipulated by introducing perspective cues. Perspective cues included modifications of shape, size, and position of the dots as a result of a perspective projection [13]. Levels of perspective manipulation (corresponding to 30%, 50%, and 70% FV responses) were determined individually for each participant during a preliminary adjustment task.

#### Preliminary adjustment task

Despite the absence of differentiating visual cues, when presented with an orthographic projection of a point-light walker observers show a strong preference to interpret the stimulus as facing towards them [12], [13], [15], [16]. To obtain subjectively bistable stimuli, we established for each participant the *point of subjective ambiguity* (PSA) [13], that is a stimulus with a specific degree of perspective manipulation which is interpreted half of times as FV and half of times as FAV.

Following Schouten and Verfaillie [13], the perspective cues carrying the information concerning the veridical orientation of the point-light figure were gradually manipulated (nine levels), from moderately facing the viewer, to absent (orthographic projection), to strongly facing away from the viewer. The nine levels of perspective information included one orthographic projection and eight distance manipulations of the convergence point, corresponding to −4, −8, −16, 16, 8, 6, 4, and 2 times the height of the walker (negative values indicate FV stimuli). This resulted respectively in field of view angles of −53, −28, −14, 14, 28, 37, 53, and 90 degrees.

On each trial, participants were asked to indicate whether the visually presented stimulus was perceived as oriented towards or away from them. They responded verbally and the experimenter pressed the corresponding key on a remote response box. Participants were instructed to respond according to their own subjective experience and it was stressed that an equal distribution of both response alternatives was not necessary. In total, each participant completed 270 trials (9 perspective levels * 30 repetitions). Individual perspective levels were determined by fitting a cumulative Gaussian function to the proportion of FV responses and selecting the 30%, 50% (point of subjective ambiguity, PSA), and 70% thresholds. The lowest and highest perspective manipulations allowed were −1 (−127 degrees) and 1 (127 degrees).

#### Procedure

Participants were tested individually in a silent and empty gym room. After the PSA was established for each participant, participants were shown subjectively bistable point-light walkers (3 s), and asked to decide which direction the walker was facing (FV or FAV). They performed the task in two conditions: a *no-movement condition*, in which they remained in front of the screen without moving, and a *walking condition*, in which they were walking continuously on a treadmill. Height and distance of the monitor in the walking condition were adjusted so that the position of the monitor relative to the head of the participants was the same as during the no-movement condition (60 cm). Before starting the task, the speed of the treadmill was adjusted for each participant so that the participant's walking speed matched the speed of the point-light walker.

To avoid short-term priming effects of the walking condition on the no-movement condition, the no–movement condition always preceded the walking condition. Each participant completed 90 trials (3 perspective levels * 30 repetitions) presented in randomized order for each condition. The whole session lasted about 50 minutes per participant.

#### Data Analysis

For each participant we calculated the proportion of FV responses in the two experimental conditions (walking and no-movement), separately for the 30%, 50%, and 70% FV levels. Participants whose mean proportion of FV responses in the no-movement condition was below .35 or above .65 were not included in the experimental sample (N = 1), because of imprecise threshold estimate (expected mean value for a perfect estimate = .50).

To aid the comparison between the different perspective levels (30%, 50%, and 70% FV), we computed for each participant and for each perspective level a *facing the viewer index* (*FVi*), defined as the ratio of FV responses for the walking condition and the no-movement condition:
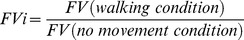
Values of this index greater than one indicate that FV responses were more frequent in the walking condition compared to the no-movement condition, whereas values lower than one indicate that FV responses were more frequent in the no-movement condition compared to the walking condition.

One-sample t-tests on the *FVi* were performed to ascertain whether the proportion of FV responses in the walking condition was significantly different from the proportion of FV in the no-movement condition, i.e., whether the *FVi* was significantly different from 1. In order to investigate the effect of perspective level, *FVi* was submitted to ANOVA, with perspective level (30%, 50%, and 70% FV) as within-subject factor.

### Experiment 2

#### Participants

Twenty-nine undergraduate and graduate students from the University of Turin (11 male and 18 female, mean age = 23.6 years, age range 19–30) volunteered to take part in the experiment. All had normal or corrected-to-normal vision, had provided informed written consent, and were naïve with respect to the purpose of the study. None of the Experiment 2 participants had participated in Experiment 1. Four participants were excluded from data analysis due to imprecise PSA estimate. Participants included in Experiment 2 did not differ from participants in Experiment 1 concerning age, gender, and mean proportion of FV responses during the no-movement condition (*p_s_*>.10).

#### Stimuli and Procedure

Stimuli, apparatus, and procedure were the same as in Experiment 1, but the walking condition was substituted by a *lateral-step condition* which did not involve the use of the treadmill. Participants were asked to start from a central position (in front of the monitor, whose height and distance were adjusted as during Experiment 1) and to make continuous rhythmic lateral steps in the horizontal plane (one step on the left, back to the center, one step on the right, and so on). This action was chosen because, as walking, it involves the rhythmic and coordinated movement of lower and upper limbs. In addition, it provides an optimal control for lateral head movements, which may affect performance in in-depth perceptual tasks. Before starting, participants were asked to adjust the rhythm of the steps to those of the point-light walker.

### Experiment 3

#### Participants

A new group of 25 students from the University of Turin (9 male and 16 female, mean age = 24.4 years, age range 21–34) participated in the experiment. All had normal or corrected-to-normal vision, had provided informed written consent, and were naïve with respect to the purpose. One participant was excluded from data analysis due to imprecise PSA estimate. Participants included in Experiment 3 did not differ from participants in Experiment 1 concerning age, gender, and mean proportion of FV responses during test-phase 1 (*p_s_*>.05).

#### Stimuli and Procedure

Stimuli, apparatus, and procedure were the same as in Experiment 1, but the walking condition was substituted by a *cycling condition*. Participants performed the task on an exercise bicycle, in front of a monitor (whose height and distance were adjusted as during Experiment 1), and were asked to pedal continuously keeping a constant speed. Before starting, participants were asked to adjust the rhythm of the cycling to the steps of the point-light walker (1 push on the pedal = 1 point-light walking step).

## Results

### Experiment 1 - Does motor activation influence the perceived orientation of observed actions?

**Figure 2:**
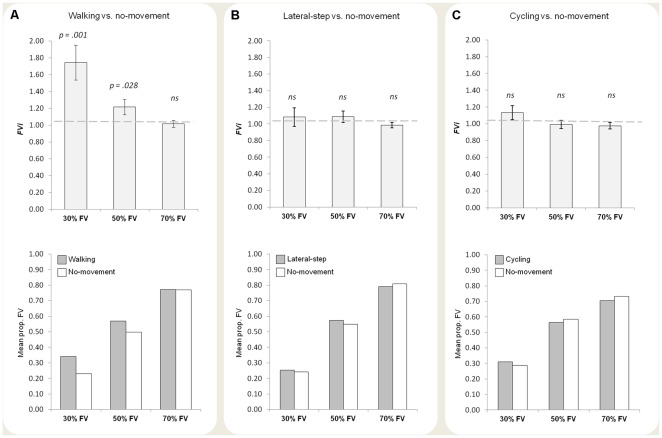
Results of the three experiments. (A) Experiment 1: walking condition vs. no-movement condition (N = 24). Upper panel. Plot of the *FVi* for the three perspective levels (30%, 50%, and 70% FV). The dashed line at 1 corresponds to the case in which FV responses were equally frequent in the walking condition and in the no-movement condition. Values greater than one indicate that FV responses were more frequent in the walking condition compared to the no-movement condition. Bars indicate standard errors. Lower panel. Mean proportion of FV responses in the walking condition (gray bars) and the no movement condition (white bars). (B) Experiment 2: lateral-step condition vs. no-movement condition (N = 25). Upper panel. *FVi* was not significantly different from one for any of the perspective levels. Lower panel. Mean proportion of FV responses in the lateral step condition (gray bars) and the no movement condition (white bars). (C) Experiment 3: cycling condition vs. no-movement condition (N = 24). Upper panel. *FVi* was not significantly different from one for any of the perspective levels. Lower panel. Mean proportion of FV responses in the cycling condition (gray bars) and the no movement condition (white bars).

In Experiment 1, the estimated PSA ranged from 3.0 to 45.8 (*M* = 8.3, *SD* = 8.4). Participants were asked to judge the in-depth orientation of ambiguous point-light walkers while walking themselves on a treadmill (walking condition) or remaining in front of the screen without moving (no-movement condition). We found that walking significantly increased the proportion of FV responses (mean *FVi* significantly different from 1, *M* = 1.32, *SD* = .38; *t*_(23)_ = 4.23, *p*<.001, *d* = .86). The mean proportion of FV responses ranged from 0.38 to 0.63 (*M* = 0.50, *SD* = 0.07) for the no-movement condition, and from 0.40 to 0.77 (*M* = 0.56, *SD* = 0.12) for the walking condition. Within-subject ANOVA revealed a significant effect of perspective level (*F*_(2,46)_ = 6.92, *p* = .005, *η^2^_p_* = .39), with the *FVi* values linearly decreasing from the 30% FV level to the 50% FV level, to the 70% FV level (linear contrast: *F*_(1,23)_ = 11.39, *p* = .003, *η^2^_p_* = .33). Orthographically projected point-light walkers (i.e., without perspective cues) are predominantly seen as facing the viewer [12], [15], [16]. These findings indicate that the effect of concurrent motor execution on the in-depth perception of the walker became more robust the more strongly perspective information supported the non-dominant facing away interpretation. In the 30% FV condition, the effect of observer movement on FV interpretations was most pronounced. In the 70% FV condition, the FV interpretation was already so strong that there simply was no room left for an effect of observer movement (Fig. 2A).

### Experiment 2 - Is the effect due to motor simulation?

In Experiment 2 the participants' walking action was substituted by lateral steps. Based on the notion of common coding [17], the degree of representational overlap between executed and observed movements should modulate the degree to which simulation processes are recruited during action observation [18]. As a consequence, the influence of action execution on the perception of biological motion should critically depend on the motor similarity between executed and observed behavior [19]–[22] and no FV responses increase should be observed when performing lateral steps. Results confirmed this prediction. The estimated PSA in the experimental sample ranged from 2.9 to 10.1 (*M* = 5.0, S*D* = 2.0). Most importantly, in contrast to Experiment 1, the mean *FVi* (*M* = 1.05, *SD* = .24) did not significantly differ from 1 (*t*_(24)_ = 1.01, *p* = .324, *d* = .20), indicating that the proportion of FV responses was similar for the no-movement and the lateral-step conditions (Fig. 2B). The mean proportion of FV responses ranged from 0.43 to 0.64 (*M* = 0.53, *SD* = 0.07) for the no-movement condition and from 0.39 to 0.71 (*M* = 0.54, *SD* = 0.10) for the lateral-step condition (Fig. 2B). No significant effect of perspective level (30%, 50%, and 70% FV) was found (ANOVA, *F*_(2,48)_ = .83, *p* = .451, *η^2^_p_* = .07).

### Experiment 3 - Is the effect due to movement direction?

A potentially relevant difference between the walking and the lateral-step condition was that participants moved *towards* (i.e., in the direction of) the point-light figure in the walking condition, but not in the lateral-step condition. Although there was no real forward progression during treadmill locomotion, it is still possible that in the walking condition participants experienced approaching the point-light figure. As approach (and avoidance) behaviors influence stimulus evaluation [23], [24], this difference could potentially be the source of the differential effect reported for walking and performing lateral steps. We addressed this issue in Experiment 3. Instead of walking on a treadmill, participants were asked to judge the in-depth orientation of bistable point-light walkers while riding an exercise bike. If the increase in FV responses for the walking condition was due to movement direction, then cycling *towards* the point-light figure should result in a similar increase in FV responses. If, however, the effect was due to motor simulation, cycling shouldn't cause an increase in the proportion of FV responses. Results clearly showed that movement direction was not the source of the effect. The mean *FVi* (*M* = 1.03, *SD* = .17) did not significantly differ from 1 (*t*_(23)_ = .95, *p* = .351, *d* = .19; Fig. 2C). The mean proportion of FV responses ranged from 0.43 to 0.62 (*M* = 0.53, *SD* = 0.05) for the no-movement condition, and from 0.40 to 0.64 (*M* = 0.53, *SD* = 0.07) for the cycling condition (Fig. 2C). The estimated PSA in the experimental sample ranged from 2.8 to 9.4 (*M* = 4.3, S*D* = 1.5). No significant effect of perspective level (30%, 50%, and 70% FV) was found (ANOVA, *F*_(2,46)_ = 1.38, *p* = .272, *η^2^_p_* = .11).

## Discussion

Body orientation modulates motor activation during observation of the actions performed by conspecifics [9], [10]. Our results show a complementary effect in the opposite direction, from motor execution to perceived body orientation: Concurrent motor execution increases the probability to perceive bistable point-light actions as facing the viewer (FV). This effect was found only when the executed action matched the observed action (Experiment 1). When executed and observed movement patterns differed, motor execution did not affect the evaluation of the observed action (Experiments 2 and 3). These findings support the hypothesis of a bi-directional link between body orientation and motor simulation processes. Actions towards the observer elicit stronger motor simulation within the observer's motor system [9], [10]. Conversely, motor simulation can itself bias the perceived orientation of the observed actions towards the observer.

A large body of evidence suggests that perception and action are intertwined and can influence each other by virtue of similarity: just as perception can influence action, action can influence perception [11]. Under varying conditions, action production has been shown to influence perceptual sensitivity to similar actions by conspecifics [19]–[21], [25]–[33]. Walking production, for example, has been shown to selectively affect walking perception in gait-speed discrimination task [29]. In a similar vein, Christensen et al. [19] demonstrated that performing waving arm movements either facilitates or interferes with concurrent visual detection of arm movements depending on the temporal synchrony and spatial congruency between executed and observed movements. Our findings add to this literature suggesting that action production can constrain and even determine the way observers perceive others' actions: Acting observers are not only more responsive to the visual stimuli that are similar to their actions [11], they tend to perceive those stimuli as oriented *towards* them.

Action-induced modulation of perception has been proposed to fulfill an important function in the context of social interaction: By priming perception of similar events, action production might render individuals selectively susceptible to actions of conspecifics that share features with one's own actions [11]. This mechanism of ‘perceptual resonance’ could be decisive for grounding empathy, sympathy, reciprocal imitation, and joint action. Our findings support the theorized social function of action-induced modulation of perception: Walking observers perceive walking actions as oriented towards them. Because actions oriented toward the viewer usually are more socially relevant than actions oriented away, this may indicate that perceptual resonance increases the social relevance of observed actions.

One limitation of the current work is the restriction to bistable *walking* actions. Research on the in-depth perception of point-light figures demonstrated perceptual bistability for other point-light actions, but also revealed that the presence of a preferred interpretation strongly depends on the specific movement features [15]. Future studies will be necessary to understand whether the concomitant motor execution influences the in-depth perception of actions other than walking and whether this influence always biases the perceived orientation of the observed actions towards the observer. Moreover, although the hypothesis of a direct link between body orientation and motor activation has been derived from research on motor simulation processes, future investigation should remain open to the possibility that differences in the visual analyses of human movement are involved in the manipulation of perception by concurrent motor execution. Measuring lateral head movements and controlling for possible differences in eye movement patterns between moving and stationary observers may help to clarify this issue [16].

In traditional laboratory studies observers remain stationary. As a result little is known about how moving observers perceive human movement. By showing that action production influences the perceived orientation of similar actions by conspecifics, our findings are directly relevant for theories of biological motion perception and, more generally, for motor theories of social cognition, as they suggest that the way humans perceive others' actions critically depends on the congruency between executed and observed behavior.
